# 

**Published:** 1916-05-13

**Authors:** 


### CONTENTS.

PAGE

**Methods Military and Medical** ... ... ... 139

**Self-Denial in a Minor Key** ... ... ... 140

**Hospital and Institutional News** ... ... ... 141
Newspapers and the War—Florence Nightingale's Opinion of Shakespeare—An Ill-Considered Request—Temperament and Family Life—Re-Examination of Rejected Recruits—Allowances for Wounded Soldiers—The Visual Standards of the British Army—German Surgical "Statistics"—The Craze for Gift Sales—A Deserving Charity—A Belated Settlement—An Extra Grant for Welsh University Education—The Shadwell Hospital Report—A Gross Anachronism—The Small Ward in Sanatoria—The Widow's Mite—The Children's Country Holiday Fund—Courage in Public Health Work—Trouble at a New Zealand Hospital—Deaths by Poisoning—The Army Council and Women Dispensers—"Printer's Pie, 1916"—This Week's Drug Market.

**Parliament and the Profession** ... ... ... 146

**Insanity and Temperament**... ... ... ... 147
II.—The Artistic Temperament.

**Passing Events and Topics** ... ... ... 148

**Peace and War** ... ... ... ... ... ... ... 149

The Surgery of Fractures.

**A Charming Medical Personality**... ... ... 150

The Late Dr. Lytton Maitland.

**Shakespeare's Medical Knowledge** ... ... 151
"One of our Greatest Masters of Medicine."

**The Book Market** ... ... ... ... ... ... 152

**Obituary** ... ... ... ... ... ... ... ... 152

PAGE

**The Matrons' and Sisters' Department** ... 153
II.—Nursing in the United States: Has Registration Protected the Uniform?—Morality, Efficiency, and Acceptability—The Spirit of Florence Nightingale—Higher Fees—Registration Helps Smaller Hospitals—An Advantage to the Public?
Round the Hospitals.
Florence Nightingale's Birthday—The College of Nursing—Miss Mary Rundle—The Smaller Training Schools—Guy's Hospital Banquet—Voluntary Labour in the Laundry.

**The School Doctor** ... ... ... ... ... 155

Defects of Hearing.

**The Incorporated Society of Trained Masseuses**... ... ... ... ... ... ... 156

**Textile Supplies for Hospitals** ... ... ... 157

XI.—Surgical Dressings.

**The Editor's Letter-Box** ... ... ... ... 159
Nurse-Training, Small Hospitals, and the College of Nursing—Hospital Construction—The Miller General Hospital.

**Hospital Results in 1915** ... ... ... ... 160
More Early Reports of the Voluntary Hospitals.

**Higher Nursing Appointments** ... ... ... vi

**St. Thomas's Hospital** ... ... ... ... vi

**Annual Meetings** ... ... ... ... ... vi

**Gifts to Hospitals** ... ... ... ... ... vi

**Institutional Needs** ... ... ... ... ... vi

**The Institutional Worker** ... ... (Suppl.) 1

Housewifery in the Army.

